# Integration of induced pluripotent stem cell-derived endothelial cells with polycaprolactone/gelatin-based electrospun scaffolds for enhanced therapeutic angiogenesis

**DOI:** 10.1186/s13287-018-0824-2

**Published:** 2018-03-21

**Authors:** Richard P. Tan, Alex H. P. Chan, Katarina Lennartsson, Maria M. Miravet, Bob S. L. Lee, Jelena Rnjak-Kovacina, Zoe E. Clayton, John P. Cooke, Martin K. C. Ng, Sanjay Patel, Steven G. Wise

**Affiliations:** 1 The Heart Research Institute, Sydney, NSW 2042 Australia; 2 Sydney Medical School, University of Sydney, Sydney, NSW 2006 Australia; 3 Royal Prince Alfred Hospital, Sydney, NSW 2042 Australia; 4 Department of Cardiovascular Sciences, Houston Methodist Research Institute, Houston, TX 77030 USA; 5 Graduate School of Biomedical Engineering, University of New South Wales, Sydney, NSW 2052 Australia

**Keywords:** Induced pluripotent stem cells, Endothelial cells, Biomaterial scaffolds, Angiogenesis, Regenerative medicine

## Abstract

**Background:**

Induced pluripotent stem-cell derived endothelial cells (iPSC-ECs) can be generated from any somatic cell and their iPSC sources possess unlimited self-renewal. Previous demonstration of their proangiogenic activity makes them a promising cell type for treatment of ischemic injury. As with many other stem cell approaches, the low rate of in-vivo survival has been a major limitation to the efficacy of iPSC-ECs to date. In this study, we aimed to increase the in-vivo lifetime of iPSC-ECs by culturing them on electrospun polycaprolactone (PCL)/gelatin scaffolds, before quantifying the subsequent impact on their proangiogenic function.

**Methods:**

iPSC-ECs were isolated and stably transfected with a luciferase reporter to facilitate quantification of cell numbers and non-invasive imaging in-vivo PCL/gelatin scaffolds were engineered using electrospinning to obtain woven meshes of nanofibers. iPSC-ECs were cultured on scaffolds for 7 days. Subsequently, cell growth and function were assessed in vitro followed by implantation in a mouseback subcutaneous model for 7 days.

**Results:**

Using a matrix of conditions, we found that scaffold blends with ratios of PCL:gelatin of 70:30 (PG73) spun at high flow rates supported the greatest levels of iPSC-EC growth, retention of phenotype, and function in vitro. Implanting iPSC-ECs seeded on PG73 scaffolds in vivo improved their survival up to 3 days, compared to cells directly injected into control wounds, which were no longer observable within 1 h. Enhanced engraftment improved blood perfusion, observed through non-invasive laser Doppler imaging. Immunohistochemistry revealed a corresponding increase in host angiogenic mechanisms characterized by the enhanced recruitment of macrophages and the elevated expression of proangiogenic cytokines vascular endothelial growth factor and placental growth factor.

**Conclusions:**

Knowledge of these mechanisms combined with a deeper understanding of the scaffold parameters influencing this function provides the groundwork for optimizing future iPSC-EC therapies utilizing engraftment platforms. The development of combined scaffold and iPSC-EC therapies could ultimately improve therapeutic angiogenesis and the treatment of ischemic injury.

**Electronic supplementary material:**

The online version of this article (10.1186/s13287-018-0824-2) contains supplementary material, which is available to authorized users.

## Background

Ischemic injury is one of the key biological events underlying the tissue pathology of some of the most debilitating diseases, including myocardial infarction, acute kidney failure, and stroke [1]. Ischemia, caused by the complete or even partial occlusion of the blood vessel network in the affected organ, results in drastic levels of nutrient and oxygen depletion (hypoxia) as well as inadequate removal of metabolic waste. Without timely and robust intervention, ischemia progresses to tissue necrosis and, in highly sensitive organs such as the brain or heart, can lead to immediate death [2, 3]. Responding to tissue ischemia, the body activates innate healing mechanisms that attempt to restore blood flow through the formation of new blood vessel networks, a process known as angiogenesis. However, these native revascularization systems often occur too slowly and/or are inadequate in magnitude to overcome large-scale ischemic injury [4]. This shortcoming has inspired a field of regenerative medicine that aims to augment and control the host angiogenic response to revascularize ischemic tissue, known as therapeutic angiogenesis.

While individual growth factors and genes regulating angiogenesis have been well characterized, their translation to therapeutic angiogenesis in vivo has been underwhelming, demonstrated by several inconclusive or negative clinical trials [5, 6]. For example, single agents such as vascular endothelial growth factor (VEGF) aiming to promote increased peripheral angiogenesis are promising in preclinical studies, but perform poorly in human trials [7]. As a result, it has become increasingly accepted that modulation of angiogenesis involves the activation of a complex network of events not readily addressed by a single growth factor or gene therapy. Thought to represent a more extensive and comprehensive control over angiogenesis, stem/progenitor cell-based therapy has gained attention due to the cells’ intrinsic characteristics as hypoxia-responsive multiparacrine release vehicles. While stem cell therapy has traditionally been plagued by inadequate cell numbers and ethical concerns regarding their origin, in recent years the advent of induced pluripotent stem cells (iPSCs) has overcome some of these barriers [8]. iPSCs can be generated from any somatic cell and possess unlimited self-renewal and differentiation potential [8]. Of the numerous iPSC-derived cell phenotypes extensively studied for proangiogenic function, iPSC-derived endothelial cells (iPSC-ECs) appear to be most promising for therapeutic angiogenesis [9–11]. However, translational challenges for iPSC-ECs common to traditional stem cell therapy remain; in particular, low levels of in-vivo engraftment and short-term viability [12]. iPSC-ECs fail to integrate with the host tissue and gradually die after delivery [9, 10, 13], highlighting an essential need for engraftment strategies capable of extending their in-vivo lifetime and potentially augmenting their proangiogenic function.

Synthetic biomaterial scaffolds have made a tremendous impact in regenerative medicine by allowing researchers to provide functional platforms containing the biophysical and chemical cues necessary to sustain stem cell behavior and function [14] that are often absent at sites of ischemic injury and necrotic tissue. The employment of biomaterial scaffolds to enhance engraftment of iPSC-ECs in vivo is a promising approach to improve therapeutic angiogenesis. However, literature surrounding iPSC-ECs combined with biomaterial scaffolds in vivo is limited, with only a few publications to date investigating this concept [15, 16]. While demonstrating enhanced angiogenic effects, these studies are conducted in immunodeficient mice that do not represent native engraftment conditions following exogenous cell injections. Additionally, the functional mechanisms underlying scaffold-mediated enhancement of iPSC-EC proangiogenic function in vivo have not yet been defined. Insight into these mechanisms is important for future optimization of combined iPSC-EC scaffold strategies and for defining the practical guidelines necessary for their improved translation.

In this study, we conduct a comprehensive analysis of electrospun polycaprolactone (PCL)/gelatin scaffolds and their potential to enhance the engraftment and proangiogenic function of iPSC-ECs in vitro and in vivo. Through tailoring of material composition and structural properties, we identify candidate scaffolds best able to support iPSC-EC growth and function in vitro. Using these scaffolds as engraftment platforms, we assess their potential to augment the proangiogenic effects of iPSC-ECs following implantation in vivo. Through gene expression and immunohistochemical analysis, we identify some of the key drivers of scaffold-mediated enhancement of iPSC-EC angiogenic function, while defining important benchmarks of scaffold performance that relate to these effects. The findings of this study provide mechanistic knowledge essential to the future development of optimized therapies combining iPSC-ECs with biomaterial scaffolds, which may ultimately improve the translation of iPSC-ECs for therapeutic angiogenesis.

## Methods

### Reagents

d-Luciferin potassium salt was purchased from Cayman Chemicals (Ann Arbor, MI, USA). Lympholyte®-M cell separation medium was purchased from Cedarlane Labs (Burlington, ON, CA). Endothelial Basal Medium (phenol red free) was purchased from Lonza (Switzerland). Polycaprolactone (PCL) polymer, gelatin, and 1,1,1,3,3,3-hexafluoro-2-proponal (HFP) solvent were purchased from Sigma-Aldrich (St. Louis, MO, USA). Purified, soluble ovine collagen was obtained from CollTech (Osborne Park, WA, Australia) and lyophilized as described previously. Multiple Stain Solution (MSS) and JB-4 resin were purchased from Polysciences Inc. (Warrington, PA, USA).

### Electrospinning

PCL and gelatin were dissolved at a total of 10% (*w/v*) in HFP in varying ratios. Solutions were loaded into a syringe and fed through a 0.6-mm-diameter needle at a flow rate of either 8 ml/h or 16 ml/h using a syringe pump. The needle was connected to a 20-kV positive power supply and directed at a grounded parallel plate collector at a distance of 20 cm. Scaffolds were then cut into circular discs using a 6-mm-diameter biopsy punch. Before cell seeding and implantation, scaffolds were sterilized by UV light for 30 min and washed three times with and stored in sterile PBS.

### Scaffold characterization

Scaffold surfaces were imaged under a 15-kV scanning electron microscope at 4000× magnification under high vacuum conditions. Images were taken for *n* = 5 scaffolds/group and then *n* = 100 fibers were measured for each sample. For porosity measurements, scaffolds were embedded in JB-4 resin and cut into 5-μm cross-sections. The cross-sections were stained using Multiple Stain Solution and imaged at 40× magnification. Cross-section images were then converted to 8-bit grayscale and the percentage of white (pores) vs black (scaffold) was quantified as porosity. For scaffold wetting experiments, scaffolds were soaked in PBS for 7 days at 37 °C. Scaffolds were then completely dried overnight in a fume hood at RT prior to scanning electron microscopy (SEM) imaging.

Mechanical testing of the electrospun scaffolds was performed on an Instron Tensile Machine (Instron 5543). Briefly, scaffolds were cut into strips of 0.5 cm × 3.5 cm and clamped into the Instron. A constant pull of 3 mm/min was applied and the force measured by a 50-N load cell. Wet samples were incubated in PBS at 37 °C for 1 h and dried prior to measurement. The elastic modulus was obtained from the linear region of the stress/strain curve. Ultimate tensile strength was defined as the maximum force at break.

Degradation testing of electrospun scaffolds was performed by immersing scaffold samples in a 1 U/ml Protease XIV (Sigma) solution at 37 °C for 4 days. Samples were preweighed prior to immersion and were allowed to dry prior to reweighing. Degradation was expressed as the percentage of initial weight remaining per day.

### iPSC-EC derivation and culture

**Fig. 1:**
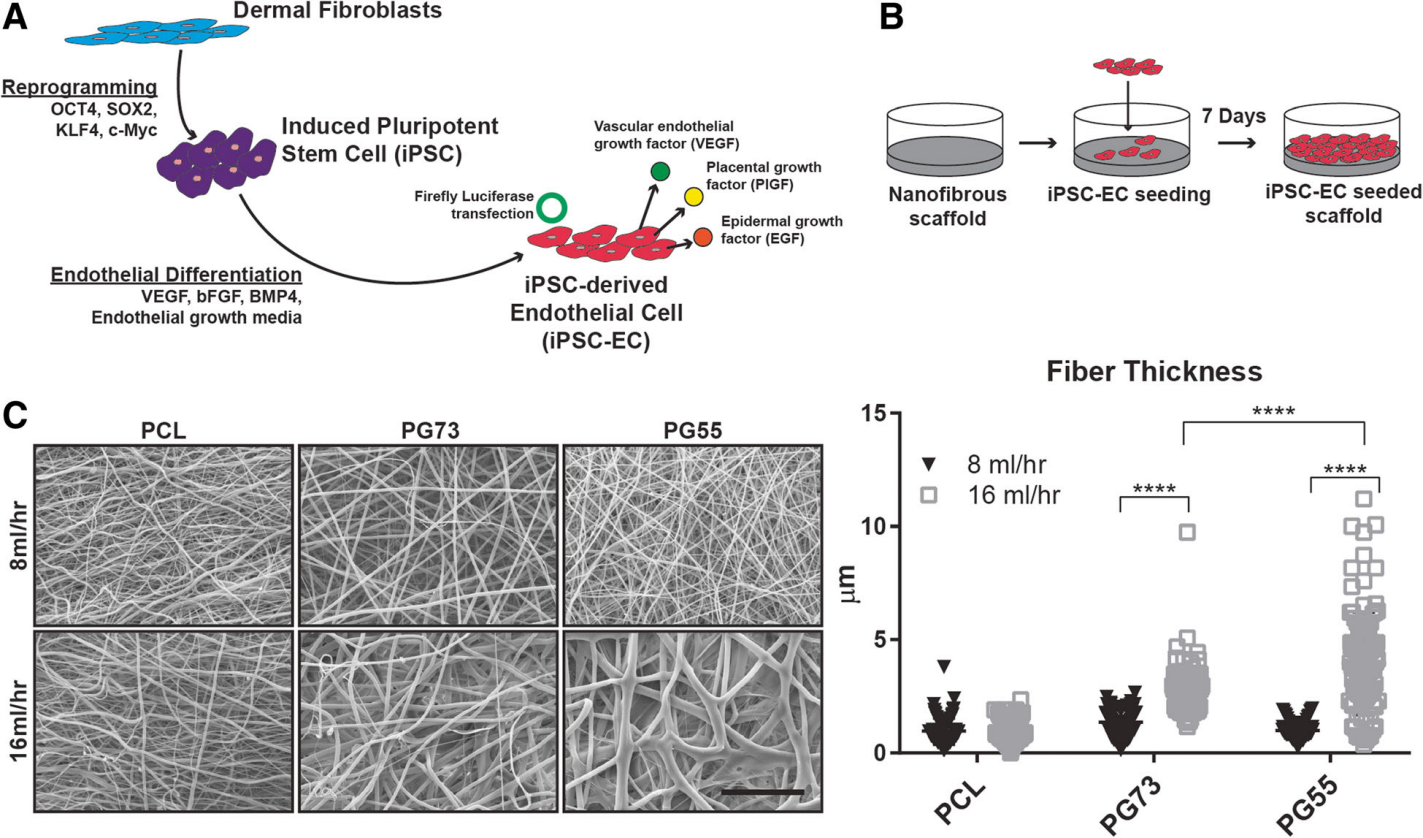
**a** Schematic of iPSC-EC differentiation and characterization. **b** iPSC-EC seeding protocol on electrospun scaffolds. **c** SEM images of PCL/gelatin scaffolds at varying flow rates and PCL/gelatin blends; quantification of fiber thickness (inset), scale bar represents 50 μm. ****p < 0.0001. *n* = 5 scaffolds/group and *n* = 100 fibers/scaffold. PCL polycaprolactone, PG73 PCL/gelatin (70:30 ratio), PG55 PCL/gelatin (50:50 ratio)

iPSC-ECs were fully differentiated and characterized, as described previously [17], prior to use in experiments. Briefly, iPSCs were generated via retroviral overexpression of Oct4, SOX2, KLF4, and c-Myc in adult human dermal fibroblasts and differentiated into endothelial cells using previously described methods [9, 18]. On day 14 of differentiation, iPSC-ECs were purified using FACS to sort for CD31^+^ cells (Anti-human CD31-PE; eBioscience Inc., San Diego, CA, USA) as described previously [19]. iPSC-ECs were also stably transfected with a reporter expression system encoding the firefly luciferase enzyme as an imaging modality. iPSC-ECs were cultured in EGM-2MV media (CC3202; Lonza Group Ltd) and seeded on PCL/gelatin scaffolds at a concentration of 1 × 10^5^ cells/scaffold in a total media volume of 200 μl. After 24 h, scaffolds were placed into fresh EGM-2MV and the media changed every other day for 7 days (Fig. 1b). For hypoxia experiments, iPSC-ECs were cultured in 1% O_2_ for 24 h prior to RNA extraction and subsequent qPCR analysis. Prior to implantation, iPSC-EC-seeded scaffolds and iPSC-ECs alone were washed and suspended in basal EGM to remove all endothelial growth factors from the media.

### Angiogenic gene expression in vitro

Quantitative PCR was performed using iQ SYBR-Green Supermix and the iCycler Real-time PCR Detection System (Bio-Rad). Gene expression was calculated using primers for vascular endothelial growth factor (VEGF) (forward, 5′-TGCCAAGTGGTCCCAG–3′; reverse, 5′-GTGAGGTCTTGATCCG-3′), epidermal growth factor (EGF) (forward, 5′-GAGGAGCACGGGAAAAGAA-3′; reverse, 5′-CCGGAGCTCCTTCACATATT-3′), and placental growth factor (PlGF) (forward, 5′-GTTCAGCCCATCCTGTGTCT-3′; reverse, 5′-TTAGGAGCTGCATGGTGACA-3′), with *B2M* as a housekeeping gene.

### Mouse subcutaneous implantation

Study approval was obtained from Sydney Local Heath District Animal Welfare Committee (protocol number 2013/050). Experiments were conducted in accordance with the Australian Code of Practice for the Care and Use of Animals for Scientific Purpose. Under 2% methoxyfluorane anesthesia, wild-type FVB/n mice had their dorsal surfaces shaved, sterilized with betadine solution, and washed clean with sterile PBS. Four 1.5-cm incisions (two rows side by side) were then cut through the skin to create four subcutaneous pockets [20]. Within each wound, control EBM media, iPSC-ECs, iPSC-EC-seeded scaffolds, or scaffold alone were transplanted. The wounds were closed with 6–0 silk sutures. Mice were then allowed to recover for at least 1 h. Mice underwent IVIS imaging over the course of 9 days when bioluminescent signals from iPSC-ECs were no longer observed. Skin wound biopsies were taken at defined histological time points.

### Bioluminescence imaging

d-Luciferin was reconstituted at a concentration of 40 mg/ml. Binding of the luciferin substrate to the luciferase enzyme results in bioluminescence, quantified using the IVIS Series preclinical in-vivo imaging system apparatus (Perkin Elmer). To induce bioluminescence, luciferin was given at a total volume of 100 μl through intramuscular administration at the implantation site. Bioluminescence was measured in units of radiance (photons/s/cm^2^/sr). All bioluminescence measurements were calculated as mean radiance within a predefined region of interest (ROI). Mean radiance measurements for each ROI were averaged amongst *n* = 5 per group using repeated measurement of the same subjects over time.

### Perfusion Doppler imaging and analysis

Post surgery, perfusion at the implantation site was measured by laser Doppler MOOR-LMD V192 (Moor Instruments, UK). Mice were anesthetized prior to imaging and the anesthetic removed during imaging. Three repeat Doppler measurements were taken after removal of the anesthetic to prevent perfusion data being affected by variations in depth of anesthesia. Doppler recordings were taken of the entire mouse back in a scan area of 6 cm × 8 cm at a scan rate of 10 px/s. Doppler measurements (*n* = 5 per group) were quantified by drawing equal areas with the surgical wound of the implantation site located in the middle of the quantification area.

### Histology and immunohistochemistry

Histology and immunohistochemistry analysis was conducted as described previously [20]. Briefly, for histology stains, standard H&E was used to assess total cell infiltration. For immunohistochemistry analysis, sections were stained with primary antibodies including anti-VEGFA (Abcam, USA) for vascular endothelial growth factor expression, anti-PlGF (Abcam, USA) for placental growth factor expression, anti-NE (neutrophil elastase) (Abcam, USA) for neutrophils, anti-CD68 (Abcam, USA) for macrophages, anti-CD31 (Abcam, USA) for endothelial cells, anti-vimentin (Abcam, USA) for fibroblasts, and anti-SMC-α actin (Abcam, USA) for smooth muscle cells. Fluorescence microscopy was then used to visualize cell markers using Alexa Fluor 594-conjugated secondary antibodies. Sections were mounted with DAPI-containing mounting media (VECTASHIELD).

### Quantitative image analysis

Quantification of bioluminescence images was performed using LivingImage 4.5 (Perkin Elmer) software. Histological and immunohistochemical sections were imaged using a Zeiss Upright Olympus fluorescence multichannel microscope, captured with a Nikon DP Controller 2.2 (Olympus, Japan). Immunohistochemical and histopathological analysis was done using ImageJ. For immunohistochemical analysis, marker expression was expressed as the positive stained area determined by a common threshold intensity divided by the tissue area of measurement. All markers were quantified from *n* = 6 sections per group per time point.

### Statistical analysis

Data are expressed as mean ± SEM and statistical significance indicated as either *p* < 0.05, *p* < 0.01, *p* < 0.001, or *p* < 0.0001. The data were compared using one-way ANOVA followed by Bonferroni’s post-hoc test or two-way ANOVA followed by Tukey’s post-hoc test using GraphPad Prism version 5.00 (GraphPad Software, San Diego, CA, USA) for PC.

## Results

### Scaffold characterization

Solutions of pure PCL and blends of PCL and gelatin at ratios of 70:30 (PG73) and 50:50 (PG55) were electrospun at low (8 ml/h) and high (16 ml/h) flow rates. SEM analysis of scaffold surfaces showed that the fiber width increased at high flow rates in gelatin-containing blends only (1.35 ± 0.53 vs 2.63 ± 1.04 μm in PG73, *p* < 0.0001; 0.98 ± 0.38 vs 3.95 ± 2.27 μm in PG55, *p* < 0.0001) (Fig. 1b). High flow rate fibers were largest in PG55 blends followed by PG73 (3.95 ± 2.27 vs 2.63 ± 1.04 μm, *p* < 0.0001; Fig. 1b). However, PG55 scaffolds had a larger distribution of fiber widths compared to PG73. SEM analysis of high flow rate PG73 and PG55 scaffolds after 7 days of wetting in PBS showed that PG55 fibers appeared to be more structurally compromised compared to PG73 fibers, indicated by a loss of fiber morphology (Additional file 1: Figure S1A).

To further examine the physical stability of PG73 scaffolds, mechanical testing and degradation experiments were conducted. Mechanical testing of PG73 scaffolds showed that wetting of the scaffold did not compromise structural integrity, indicated by no significant changes in ultimate tensile strength (UTS) and Young’s modulus before and after wetting (Additional file 2: Figure S2B, C). Additionally, under accelerated degradation conditions in a Protease XIV solution at 37 °C for 4 days, PG73 scaffolds showed similar degradation profiles as pure PCL (Additional file 2: Figure S2D).

Cross-sectional imaging revealed the internal structure of scaffolds, allowing for an estimation of pore size. No significant differences were observed between scaffold groups; however, pore size tended to be reduced with both higher flow rates and increasing gelatin content (Additional file 1: Figure S1B).

### iPSC-EC growth and phenotype on scaffolds

**Fig. 2:**
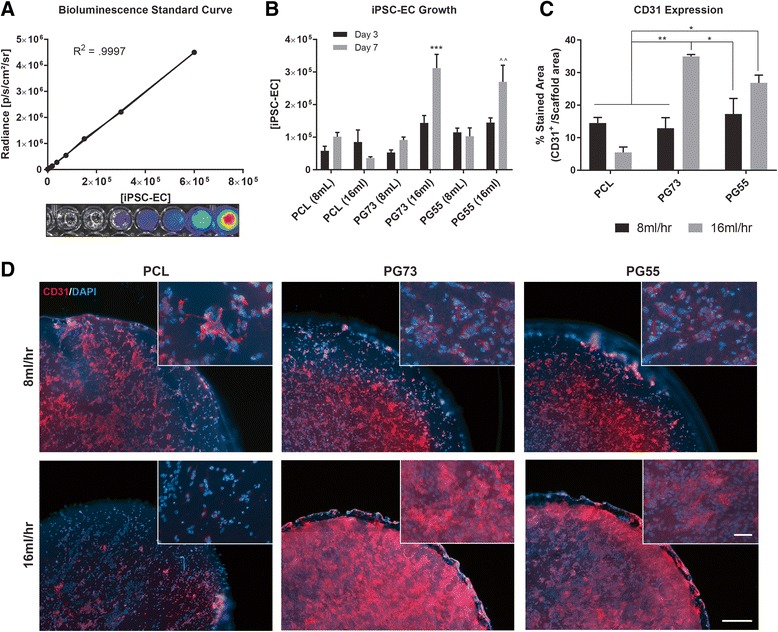
**a** Bioluminescence standard curve of iPSC-EC cell number, representative cell bioluminescence images (inset). **b** iPSC-EC growth at 3 and 7 days on electrospun scaffolds of varying flow rate and PCL/gelatin blends. ***p < 0.001, **p < 0.01 compared to PCL (8 ml/h) control scaffolds within the same time point. *n* = 5 scaffolds/group. **c** CD31 quantification of iPSC-EC cultures on scaffolds at 7 days *in vitro*. *p < 0.05, **p < 0.01. *n* = 5 scaffolds/group. **d** Representative photographs of iPSC-EC cultures stained with CD31 (red) and counterstained with DAPI (blue) at 5×; magnified images at 10× (inset). Scale bar represents 100 μm, inset scale bar represents 10 μm. iPSC-EC induced pluripotent stem cell-derived endothelial cell, PCL polycaprolactone, PG73 PCL/gelatin (70:30 ratio), PG55 PCL/gelatin (50:50 ratio), DAPI 4',6-diamidino-2-phenylindole

iPSC-ECs were seeded on scaffolds and cell growth was quantified using a transfected bioluminescence reporter. Generation of a standard curve demonstrated a linear correlation of bioluminescence intensity to iPSC-EC number (Fig. 2a). The detectable range of iPSC-EC bioluminescence was found to be between 1 × 10^3^ and 1 × 10^6^ cells/well (Additional file 3: Figure S3). Concentrations of iPSC-ECs less than 1 × 10^3^ cells/well were not significantly different from background signals, while those greater than 1 × 10^6^ cells/well saturated the bioluminescence reading capacity of the IVIS imaging apparatus.

At day 3 in culture, the highest cell numbers were observed on PG73 and PG55 scaffolds at high flow rates (1.44 ± 0.45 and 1.45 ± 0.28 × 10^5^ cells, respectively; Fig. 2b). By day 7, these conditions were both significantly increased compared to all scaffold groups (3.12 ± 0.73 and 2.69 ± 1.02 × 10^5^ cells, respectively, *p* < 0.001 and *p* < 0.01; Fig. 2b).

Immunofluorescence analysis using the CD31 endothelial cell marker at day 7 in culture revealed the highest CD31 expression for iPSC-ECs seeded on PG73 scaffolds at high flow rate (3.49 ± 0.11%, *p* < 0.01; Fig. 2c), followed by PG55 scaffolds at high flow rate (2.68 ± 0.41%, *p* < 0.05; Fig. 2c), when compared to PCL.

Cross-sectional staining of seeded scaffolds using DAPI nuclei counterstain revealed no differences in infiltration of iPSC-ECs into the scaffolds, irrespective of fiber size or composition at day 7 in culture (Additional file 2: Figure S2).

The elevated growth rates and high CD31 expression suggested an advantage for using PG73 high flow rate scaffolds as candidate engraftment platforms for subsequent in-vivo studies.

### Preimplantation characterization of iPSC-EC-seeded PG73 scaffolds

**Fig. 3:**
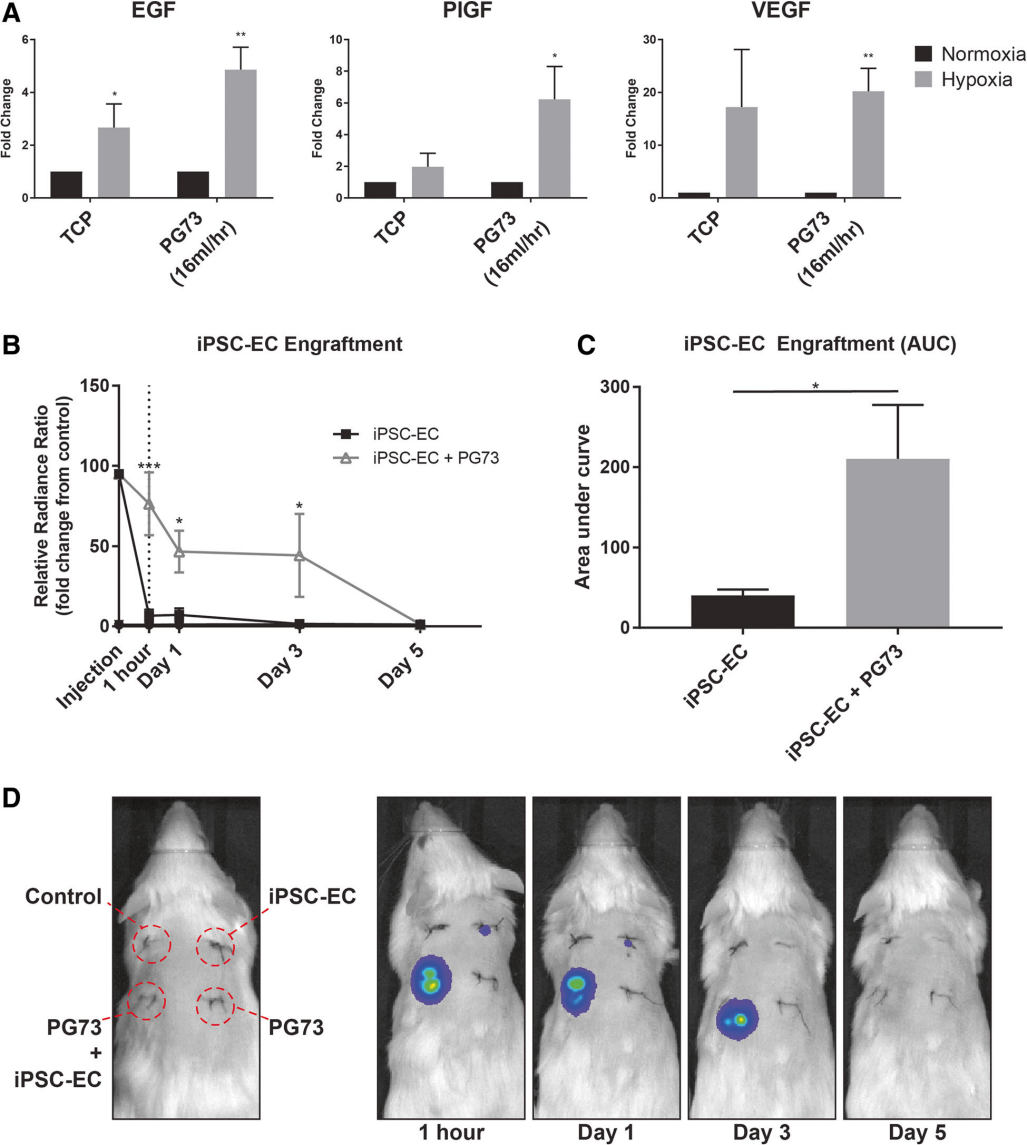
**a** Angiogenic cytokine gene expression of iPSC-ECs cultured on tissue culture plastic (TCP) and PG73 (16 ml/h scaffolds) in normoxia vs hypoxia. Values presented as fold change normalized to normoxia. *p < 0.05, **p < 0.01. *n* = 5 samples/group. **b** In-vivo engraftment curves measured through IVIS bioluminescence measurement of iPSC-ECs and iPSC-EC-seeded PG73 scaffolds over 5 days. ***p < 0.001, *p < 0.05. *n* = 5 samples (animals)/group. **c** Area under the curve quantification of engraftment bioluminescence curves. *p < 0.05. *n* = 5 samples (animals)/group. **d** Representative bioluminescence photographs of animals implanted with experimental groups over 5 days. EGF epidermal growth factor, PlGF placental growth factor, VEGF vascular endothelial growth factor, PG73 polycaprolactone/gelatin (70:30 ratio), iPSC-EC induced pluripotent stem cell-derived endothelial cell, AUC area under the curve

To assess functional changes of iPSC-ECs seeded on PG73 scaffolds after 7 days in culture, we analyzed hypoxia-induced gene expression and scaffold remodeling effects. When grown on tissue culture plastic under hypoxic conditions, iPSC-ECs upregulated the expression of epidermal growth factor (EGF) and placental growth factor (PlGF), while vascular endothelial growth factor (VEGF) upregulation was highly variable. However, when cultured on PG73 scaffolds under similar hypoxic conditions, this upregulation was further enhanced by 1.8-fold, 3.17-fold, and 1.17-fold, respectively (4.86 ± 0.85 vs 2.67 ± 0.89-fold, 6.24 ± 2.01 vs 1.97 ± 0.86-fold, and 20.25 ± 4.28 vs 17.24 ± 10.87-fold, respectively, *p* < 0.05, *p* < 0.01; Fig. 3a). The hypoxia-responsive function of iPSC-ECs on PG73 scaffolds was enhanced compared to normal tissue culture conditions.

To assess dedifferentiation of iPSC-ECs back to dermal fibroblast phenotypes, iPSC-EC cultures on PG73 and PCL scaffolds were assessed for fibroblast phenotypes compared to human dermal fibroblasts (Additional file 4: Figure S4). Endothelial and fibroblast phenotypes were assessed using the CD31 and Vimentin markers, respectively. iPSC-ECs cultured on PCL scaffolds showed a 4.1-fold decrease in CD31 expression (Additional file 4: Figure S4B) and a 2.3-fold increase in fibroblast phenotype (Additional file 4: Figure S4C). These results suggest that iPSC-ECs maintain their endothelial phenotypes when cultured on PG73 scaffolds and dedifferentiate back to fibroblast phenotypes on PCL scaffolds.

### In-vivo engraftment of transplanted iPSC-ECs and iPSC-EC PG73 scaffolds

Using our bioluminescence standard curve, we quantified 7-day iPSC-EC cell numbers on PG73 scaffolds at concentrations of ~ 3 × 10^5^ cells/scaffold. For subsequent in-vivo experiments, we used matched cell concentrations for iPSC-EC alone injections. Bioluminescence was also used to quantify subsequent engraftment and survival of iPSC-ECs in vivo. Within 1 h following implantation, iPSC-ECs alone observed an 11.42-fold decrease in survival compared to iPSC-ECs cultured on PG73 scaffolds (6.69 ± 3.73 vs 76.41 ± 19.56 radiance ratio, *p* < 0.001; Fig. 3b). Area under the curve analysis revealed a 5.25-fold increase in total engraftment of iPSC-ECs seeded on PG73 scaffolds compared to injection alone (210.3 ± 67.22 vs 40.4 ± 16.16 radiance ratio, *p* < 0.05; Fig. 3c). The magnitude and length of engraftment of iPSC-ECs on PG73 scaffolds was significantly increased compared to injection alone. Bioluminescence was no longer observed in either group after day 5.

### Blood perfusion

**Fig. 4:**
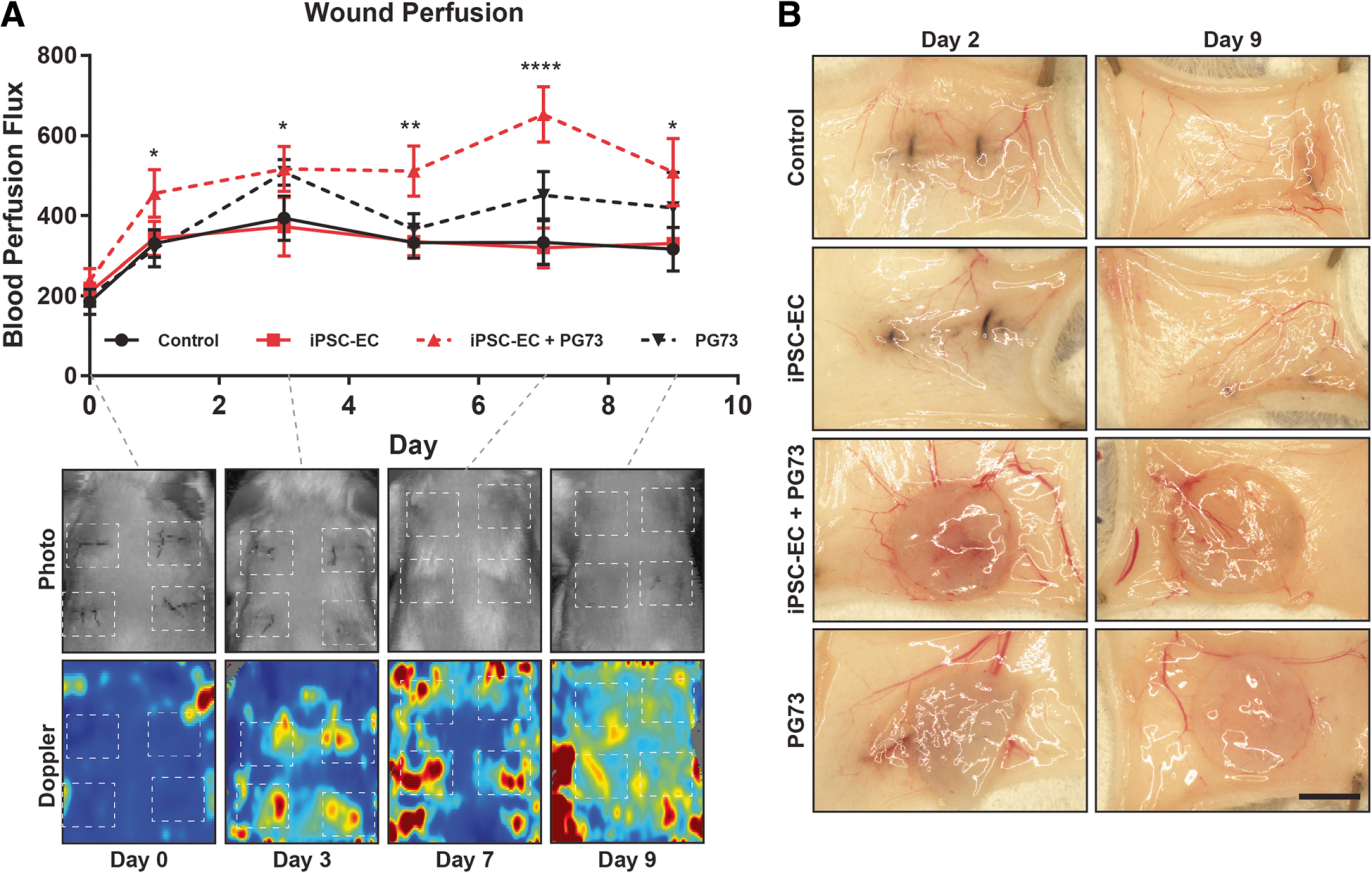
**a** Laser Doppler blood perfusion curves of areas encompassing wound site, representative photographs and Doppler images (inset). *p < 0.05, **p < 0.01, ***p < 0.001. *n* = 5 samples (animals)/group. **b** Representative macroscopic photographs of wound explants at day 2 and day 9, scale bar represents 3 mm. PG73 polycaprolactone/gelatin (70:30 ratio), iPSC-EC induced pluripotent stem cell-derived endothelial cell

Serial Doppler measurements of the implantation sites were taken to non-invasively assess blood reperfusion following tissue injury. Repeated measures analysis of the perfusion curves over 9 days revealed a significant increase in blood perfusion in the tissue surrounding iPSC-EC-seeded PG73 scaffolds compared to control wounds (Fig. 4a). The largest difference in perfusion was observed on day 7 where iPSC-EC-seeded PG73 scaffolds exhibited a 2.04-fold increase compared to control wounds (6.53 ± 0.69 vs 3.20 ± 0.49 × 10^2^ flux units, *p* < 0.0001; Fig. 4a). The implantation of PG73 scaffolds alone appeared to also increase wound perfusion, although this was not significant from control wounds.

Wound explants macroscopically appeared to show increased vasculature surrounding both iPSC-EC-seeded and acellular PG73 scaffolds. Increased vascularity was observed contacting iPSC-EC-seeded scaffolds, whereas in PG73 scaffolds the vasculature was localized to the scaffold periphery (Fig. 4b).

### Functional angiogenesis

**Fig. 5:**
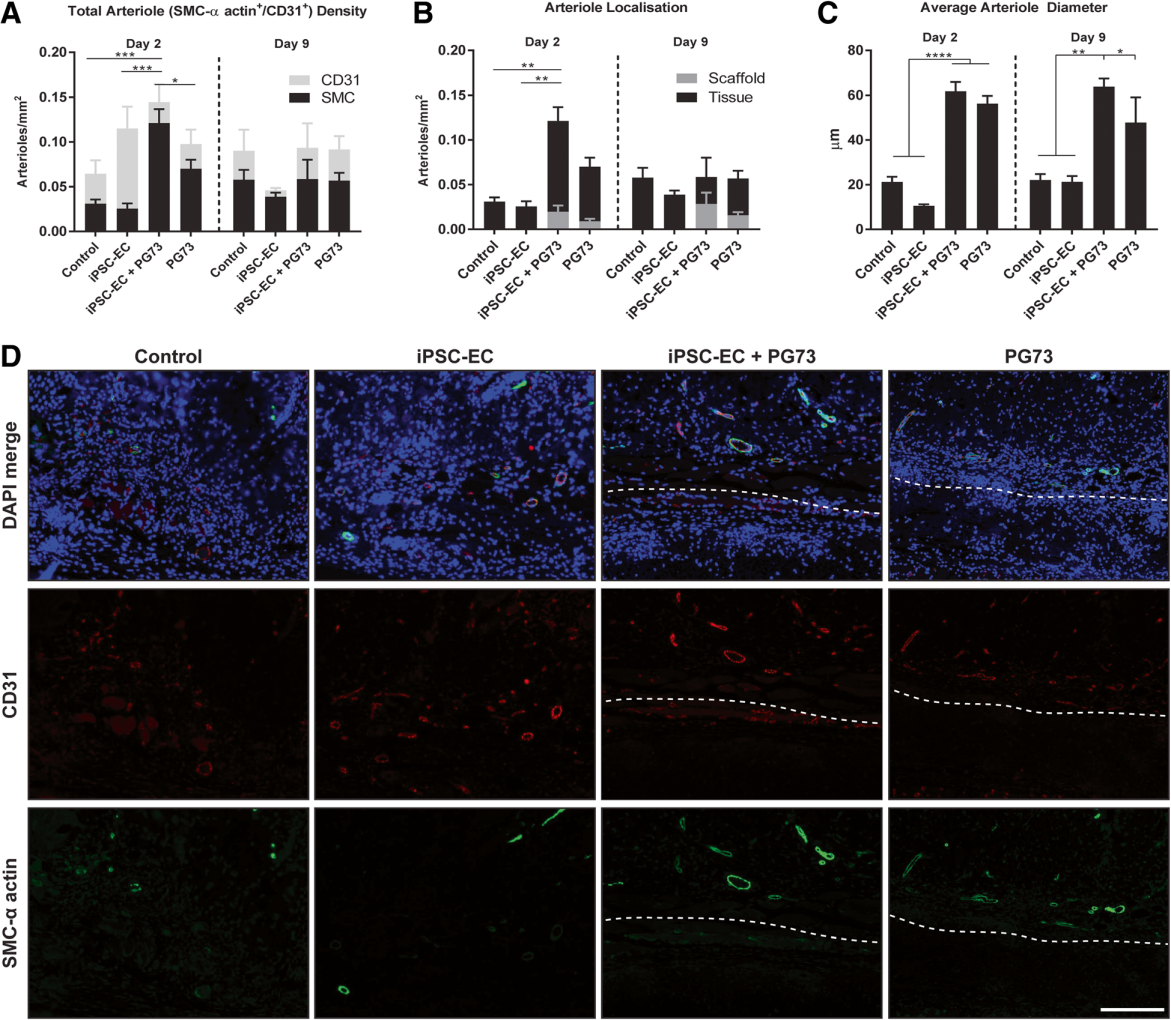
**a** Functional angiogenesis quantification of arteriole density (vessels double stained for CD31^+^ and SMC-α actin^+^). *p < 0.05, ***p < 0.001. *n* = 5 samples/group. **b** Quantification of arteriole location in surrounding tissue vs inside scaffold. **p < 0.01. *n* = 5 samples/group. **c** Quantification of average arteriole diameter. ***p < 0.001, **p < 0.01, *p < 0.05. *n* = 5 samples/group. **d** Representative photographs of DAPI/CD31/SMC-α actin merge, CD31, and SMC-α actin, respectively, at the wound site or tissue implant interface (dotted lines: above represents tissue, below represents scaffold) at day 2, scale bar represents 100 μm. SMC-α smooth muscle cell-actin, CD31 cluster of differentiation 31 (endothelial marker), PG73 polycaprolactone/gelatin (70:30 ratio), iPSC-EC induced pluripotent stem cell-derived endothelial cell, DAPI 4',6-diamidino-2-phenylindole

To determine the extent of functional angiogenesis at the implantation site, immunohistochemical analysis was conducted by double staining of SM-α actin (smooth muscle cell) and CD31 (endothelial cell) markers. Quantification of total arteriole density, identified as vessels positive for CD31 and SM-α actin (CD31^+^/SM-α actin^+^), revealed a 4.65-fold increase in iPSC-EC-seeded PG73 scaffolds compared to control, iPSC-EC alone, and PG73 alone at day 2 (0.12 ± 0.01 vs 0.03 ± 0.01, 0.03 ± 0.01, and 0.07 ± 0.01 arterioles/mm^2^, respectively, *p* < 0.05, *p* < 0.001; Fig. 5a). Arteriole localization analysis showed that this increased arteriole density was localized to the tissue surrounding iPSC-EC-seeded PG73 scaffolds (Fig. 5b). Arteriole analysis at day 9 showed no observable differences amongst all groups. Furthermore, the diameter of arterioles present in both scaffold groups, iPSC-EC-seeded PG73 scaffolds and PG73 scaffolds, were significantly increased compared to control wounds (61.86 ± 4.05 and 56.27 ± 3.49 vs 21.24 ± 2.29 μm, respectively, *p* < 0.0001; Fig. 5c). At day 9, this trend remained; however, arteriole diameters in iPSC-EC-seeded PG73 scaffold groups were even greater than in PG7 scaffolds when both were compared to control wounds (63.76 ± 3.68 and 47.86 ± 11.25 vs 22.1 ± 2.7 μm, *p* < 0.01, *p* < 0.05; Fig. 5c).

### Local innate immune response

**Fig. 6:**
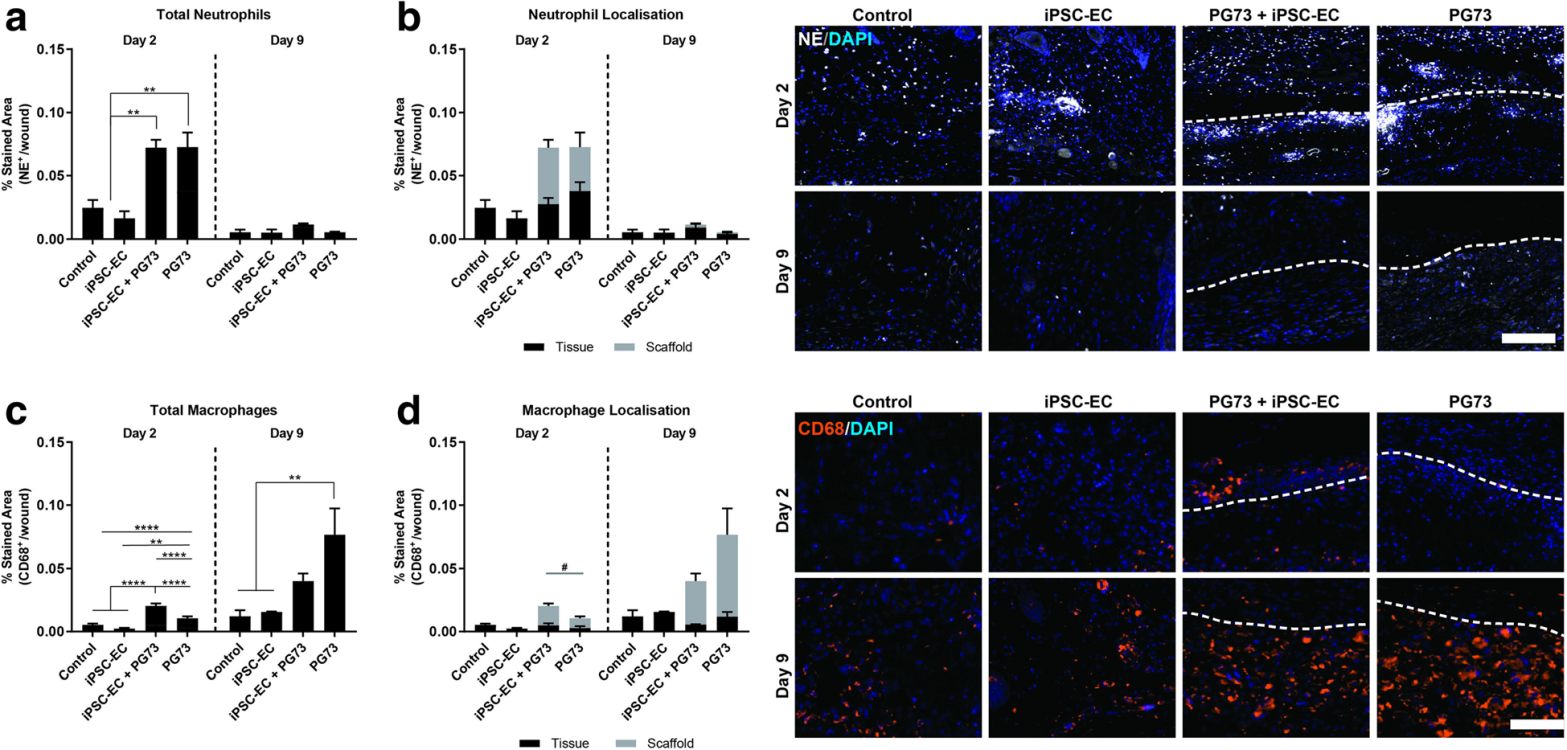
**a** Quantification of total neutrophil levels at wound site. **p < 0.01. *n* = 5 samples/group. **b** Quantification of neutrophil location in surrounding tissue vs within scaffold. **c** Quantification of total macrophage levels at wound site. **p < 0.01, ****p < 0.0001. *n* = 5 samples/group. **d** Quantification of macrophage location in the surrounding tissue vs within the scaffold. *p* < 0.05 comparing macrophages within scaffolds only. *n* = 5 samples/group. Tissue implant interface marked by dotted lines: above represents tissue, below represents scaffold. NE neutrophil elastase, iPSC-EC induced pluripotent stem cell-derived endothelial cell, PG73 polycaprolactone/gelatin (70:30 ratio), DAPI 4',6-diamidino-2-phenylindole

Local innate immune response following implantation was assessed through immunohistochemical analysis of neutrophils and macrophages using the neutrophil-elastase and CD68 markers, respectively. Scaffold implantation (both iPSC-EC-seeded and scaffolds alone) induced a 3.5-fold increase in neutrophil response compared to control wounds 2 days following implantation (~ 0.07 ± 0.01 vs 0.02 ± 0.01 stained area, *p* < 0.001; Fig. 6a). Additionally, iPSC-EC seeding did not further enhance neutrophil responses to PG73 scaffolds (0.07 ± 0.01 vs 0.07 ± 0.02 stained area; Fig. 6b). By day 9, neutrophil levels were equally resolved amongst all groups. Macrophage analysis revealed a 2-fold increase in PG73 scaffolds alone compared to control wounds (1.05 ± 0.05 vs 0.54 ± 0.01 × 10^−2^ stained area, *p* < 0.0001; Fig. 6c). iPSC-EC seeding further enhanced this macrophage response (2.04 ± 0.03 vs 0.54 ± 0.01 × 10^−2^ stained area, *p* < 0.0001; Fig. 6c). Analysis of macrophage localization showed an increased presence of macrophages within iPSC-EC-seeded PG73 scaffolds compared to PG73 scaffolds alone (1.5 ± 0.2 vs 0.8 ± 0.2, *p* < 0.01; Fig. 6d). At day 9, macrophage numbers in PG73 scaffolds were increasing, while in iPSC-EC-seeded scaffolds they appeared to be resolving (Fig. 6d).

### Angiogenic cytokine modulation and localization

**Fig. 7:**
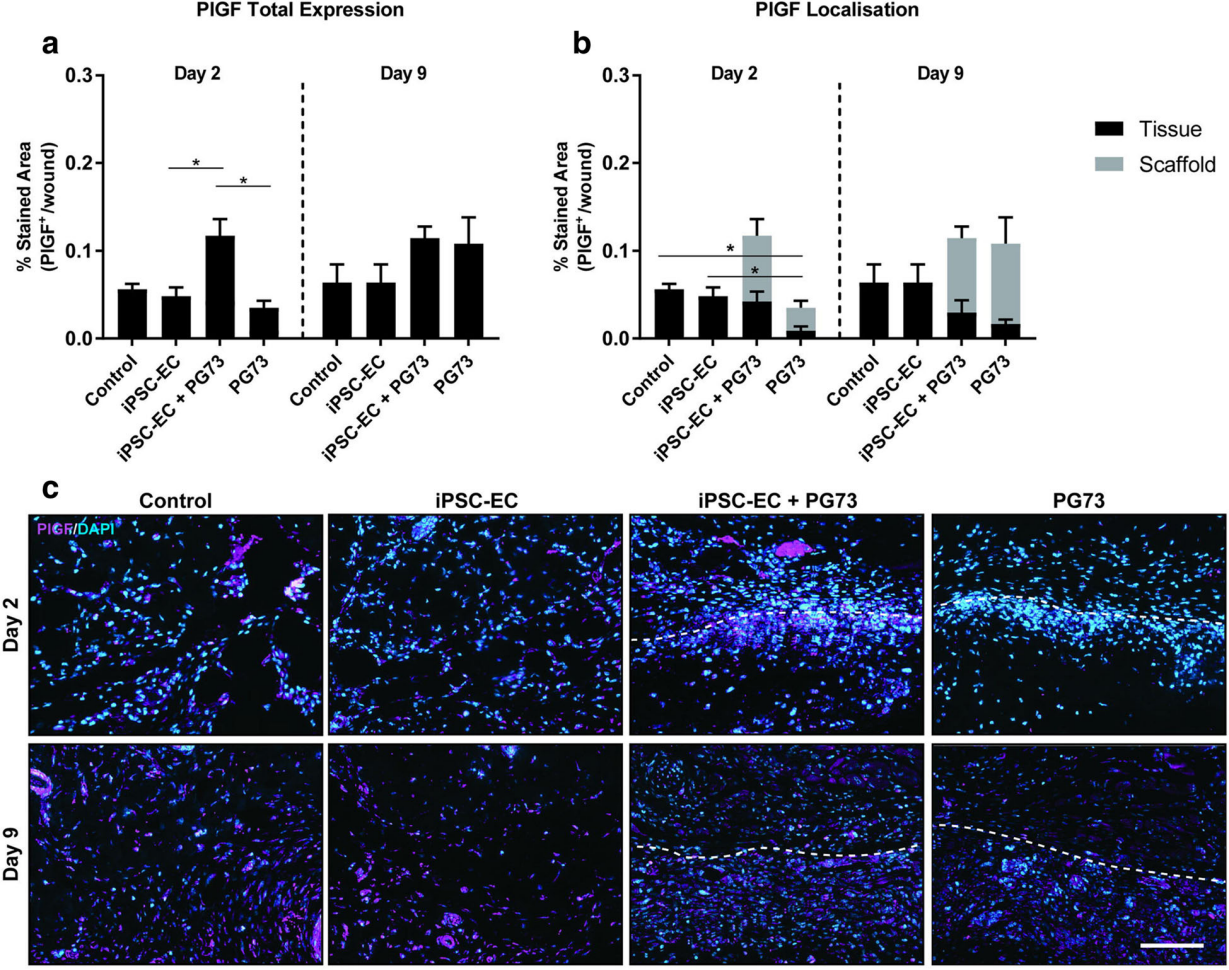
**a** Quantification of total PlGF expression at the wound site. *p < 0.05. *n* = 5 samples/group. **b** Quantification of PlGF expression location in surrounding tissue vs within the scaffold. *p < 0.05 comparing PlGF expression within the surrounding tissue only. *n* = 5 samples/group. **c** Representative photographs of PlGF staining with DAPI counterstain, scale bar represents 100 μm. Tissue implant interface marked by dotted lines: above represents tissue, below represents scaffold. PlGF placental growth factor, iPSC-EC induced pluripotent stem cell-derived endothelial cell, PG73 polycaprolactone/gelatin (70:30 ratio), DAPI 4',6-diamidino-2-phenylindole

Angiogenic cytokine analysis at the implantation site was determined using immunohistochemical analysis of the classic proangiogenic cytokines placental growth factor (PlGF) and vascular endothelial growth factor (VEGF). At day 2, significant upregulation of total PlGF was observed in iPSC-EC-seeded PG73 scaffolds compared to control, iPSC-EC alone, and PG73 alone (0.11 ± 0.01 vs 0.06 ± 0.01, 0.05 ± 0.01, and 0.03 ± 0.01 stained area, respectively, *p* < 0.05; Fig. 7a). Localization analysis showed that PG73 scaffold implantation alone decreased the baseline levels of PlGF found in control wounds (0.01 ± 0.01 vs 0.06 ± 0.01 stained area, *p* < 0.05; Fig. 7b) At day 9, no differences in PlGF total or localization were observed.

**Fig. 8:**
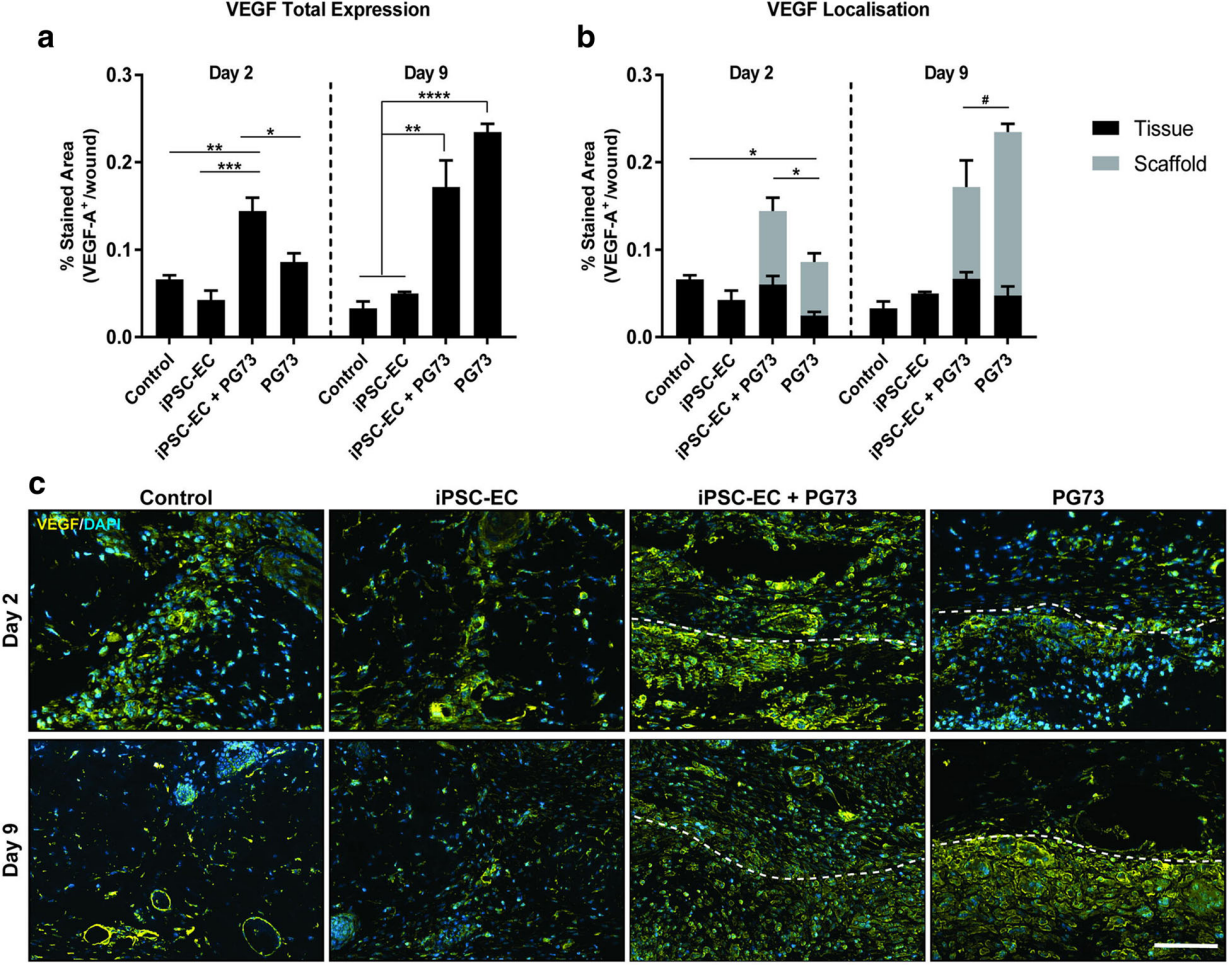
**a** Quantification of total VEGF expression at the wound site. *p < 0.05, **p < 0.01, ***p < 0.001, ****p < 0.0001. *n* = 5 samples/group. **b** Quantification of VEGF expression location in surrounding tissue vs within the scaffold. *p < 0.05 comparing VEGF expression within the surrounding tissue only, #*p* < 0.05 comparing VEGF expression within scaffolds only. *n* = 5 samples/group. **c** Representative photographs of VEGF staining with DAPI counterstain, scale bar represents 100 μm. Tissue implant interface marked by dotted lines: above represents tissue, below represents scaffold. VEGF vascular endothelial growth factor, iPSC-EC induced pluripotent stem cell-derived endothelial cell, PG73 polycaprolactone/gelatin (70:30 ratio), DAPI 4',6-diamidino-2-phenylindole

Analysis of VEGF expression at day 2 showed a significant upregulation of VEGF in iPSC-EC-seeded PG73 scaffolds compared to control, iPSC-EC alone, and PG73 alone (0.14 ± 0.01 vs 0.07 ± 0.01, 0.04 ± 0.01, and 0.08 ± 0.01 stained area, respectively, *p* < 0.05, *p* < 0.01, *p* < 0.001; Fig. 8a). Localization analysis revealed that PG73 scaffolds alone resulted in a decrease of VEGF in the surrounding tissue compared to iPSC-EC-seeded PG73 scaffolds and control wounds (0.03 ± 0.01 vs 0.07 ± 0.01 and 0.06 ± 0.01 stained area, *p* < 0.05; Fig. 8b). At day 9, total VEGF expression was significantly increased in PG73 scaffolds alone and iPSC-EC-seeded PG73 scaffolds compared to control wounds (0.17 ± 0.03 and 0.24 ± 0.01 vs 0.03 ± 0.01 stained area, respectively, *p* < 0.001, *p* < 0.0001; Fig. 8a). Between the groups, the highest expression of VEGF was observed in PG73 scaffolds alone. Furthermore, this increased VEGF expression was localized to within PG73 scaffolds (0.19 ± 0.01 vs 0.11 ± 0.03 stained area, *p* < 0.05; Fig. 8b).

## Discussion

Induced pluripotent stem cell-derived endothelial cells (iPSC-ECs) have been identified as a promising cell population with potential for increasing therapeutic angiogenesis. Derived from iPSCs possessing unlimited sources and renewal capacity, iPSC-ECs have demonstrated regenerative properties consistent with their stem cell origins. Typically generated from patient-specific cell harvests, iPSC-derived cell therapies mitigate major cell therapy concerns regarding immunogenicity and subsequent cell rejection. Additionally, the advent of iPSC banking has paved the way for the delivery of nonautologous iPSCs without evoking immune rejection [21], thereby reducing the potential time, cost, and invasiveness of iPSC-EC therapy. However, extending the collective translational benefits of iPSC-ECs for therapeutic angiogenesis requires engraftment strategies that can extend their lifetime and maintain their function in vivo.

Using a simple PCL/gelatin composite scaffold system, we assessed the influence of fiber width and gelatin content on iPSC-EC growth and function due to the well-characterized history of these synthetic and natural materials [22, 23]. The stable transfection of luciferase into our iPSC-ECs facilitated both accurate quantification of cell numbers and the capacity for non-invasive imaging in vivo. Using a matrix of conditions including increasing gelatin content and fiber width, we determined that gelatin-containing scaffolds electrospun at high flow rates supported the highest rates of iPSC-EC growth. This is consistent with previous reports of the upregulation of gelatin-binding integrins, such as α_5_β_1_, on the surface of ECs differentiated from iPSCs [24]. When combined with increased fiber widths, gelatin-based microfiber scaffolds provided greater cell surface area for integrin-mediated signaling, a key facilitator of growth and proliferation [22]. However, of the two gelatin blends, PG73 promoted higher expression of CD31, suggesting a greater conservation of iPSC-EC endothelial cell phenotype. This effect may have been driven by the inherent structural features of nanofibrous topographies that enable the formation of cellular architectures thought to recapitulate native morphology and phenotype [25]. However, with high concentrations of gelatin the structural integrity of fibers become increasingly compromised due to heightened water solubility and mechanical weakening [23]. Despite the availability of crosslinking methods to resolve this issue, these approaches rely on cytotoxic reagents which impair scaffold function for cell delivery applications [26]. Alternatively, blends with synthetic polymers possessing high mechanical strength and hydrophobicity such as PCL are employed to overcome these structural shortcomings. However, while pure PCL scaffolds may possess greater mechanical stability, they lack the appropriate cell binding domains for optimal cellular interaction. Ideal blends consisting of appropriate amounts of PCL and gelatin, such as those observed in PG73 blends, are often required to effectively balance cell adhesion/proliferation and structural integrity, respectively. While the levels of gelatin content in PG73 scaffolds are capable of supporting iPSC-EC growth, they do not appear to negatively impact the structural integrity or degradation characteristics of the scaffold.

Characterization of iPSC-EC-seeded PG73 scaffolds prior to implantation showed that scaffolds preserved the iPSC-EC phenotype and function after 7 days in culture. The possibility of dedifferentiation to original dermal fibroblast phenotypes appears limited given the lack of fibroblast marker expression [27, 28]. Combined with high CD31 expression, this suggests that the iPSC-EC endothelial cell phenotype is best preserved on PG73 scaffolds. Furthermore, hypoxia-induced gene expression was enhanced on PG73 scaffolds when compared to conventional 2D tissue culture surfaces. This functional response is likely more representative of their in-vivo actions because they assume more native-like in-vivo morphologies on 3D surfaces compared to 2D. This enhanced functional response is highly promising for augmenting in-vivo angiogenesis as the upregulation of EGF, PlGF, and VEGF is classically associated with underlying host angiogenic mechanisms [29–31]. Taken together, the provision of simple scaffold structural and signaling cues greatly enhanced the growth of iPSC-ECs, while preserving their phenotype and promoting the expression of key proangiogenic factors. This suggests that the development of more nuanced and complex scaffold designs could provide further functional enhancements and cell survival in future studies.

We next examined the functional effects of PG73 scaffolds on iPSC-ECs in vivo, tracking cell survival and retention using non-invasive imaging of the luciferase cell reporter. The administration of iPSC-ECs alone resulted in poor survival consistent with the weight of clinical findings for traditional stem cell therapy [8]. Most cells were lost within 1 h of injection. These results are more closely representative of native engraftment conditions, compared to previous investigations of iPSC-EC function which require the use of severe combined immunodeficient (SCID) mice to enhance cell engraftment [15, 16]. The use of PG73 scaffolds extended the in-vivo lifetime of transplanted iPSC-ECs by improving structural support and providing signaling cues in the wounded area. Nanofibrous scaffolds offer a 3D platform that provides some mimicry of the in-vivo structural environment, increasing the likelihood of iPSC-EC engraftment*.* While gelatin-based nanofibrous scaffolds extend the in-vivo lifetime of transplanted iPSC-ECs up to 3 days, future biomaterial strategies addressing additional factors impairing cell engraftment, including host inflammatory responses [32] and inadequate nutrient/growth factor cues [33], may help to encourage iPSC-EC in-vivo growth and further enhance engraftment. For example, enhancements in scaffold porosity [34] or the provision of growth factors [35] may prove beneficial.

Indeed, we have determined previously that human iPSC-ECs are sensitive to the diameter and alignment of nanofibrillar collagen scaffolds [16]. Aligned nanofibrillar collagen fibrils (30 nM in diameter) guide cellular organization, modulate endothelial inflammatory response, and enhance cell survival after implantation in normal and ischemic tissues. Human iPSC-ECs cultured on aligned scaffolds persisted for over 28 days, as assessed by bioluminescence imaging, when implanted into the ischemic murine hindlimb of NOD-SCID mice [16, 17]. By contrast, ECs implanted on scaffolds without nanopatterning generated no detectable bioluminescent signal by day 4 in either normal or ischemic tissues [36].

To assess the functional effects of increased cell survival, we first used laser Doppler imaging to non-invasively quantify in-vivo blood perfusion. Perfusion for PG73 scaffolds alone, while not significantly improved compared to controls, showed some improvements, an effect previously demonstrated by other acellular biomaterial platforms [37, 38]. These previous studies suggest that altered tissue remodeling arising from the introduction of foreign materials coupled with the highly invasive nature of native endothelial cells following ischemic injury influence proangiogenic conditions. Our findings demonstrate that further scaffold supplementation with iPSC-ECs significantly augments scaffold-mediated perfusion. Macroscopically, the enhanced Doppler signal appeared to correlate well with the observation of increased vasculature around the scaffolds at explant, particularly for the iPSC-EC-seeded PG73 scaffolds. To confirm this observation, we used immunohistochemistry to examine the density of arteriole vessels as a marker for functional angiogenesis.

Arterioles are more stable blood-carrying vessels compared to capillaries [39] and their diameter is directly connected to the volume of blood flow through the vessel [40]. PG73 scaffolds alone induced the formation of some large-diameter arterioles, explaining the modest increases in perfusion observed through Doppler imaging. The further increase in the density of these large-diameter arterioles at early time points correlated well with the greater perfusion levels of iPSC-EC-seeded PG73 scaffolds. The functional enhancement of angiogenesis in the presence of iPSC-ECs is consistent with previous reports showing acute benefits in a hindlimb ischemia model and *in vitro* [11]. To gain further insights into the mechanisms underpinning this effect, we quantified some of the key angiogenic regulators.

iPSC-EC scaffold-mediated augmentation of host angiogenesis is a complex process regulated by innate immune cells and proangiogenic cytokines. Observed increases in arteriole diameter following implantation surgery arise from vasodilation of nearby blood vessels allowing increased permeability of innate immune cells responding to the material implant [41]. This characteristic foreign body response is hallmarked by the increased presence of neutrophils and macrophages around the implant [42]. While an elevated host-inflammatory response to exogenous cell delivery is common, it does not appear to be the driving force behind increased angiogenic activity surrounding iPSC-EC-seeded scaffolds in this study. Equal levels of neutrophils were observed in both iPSC-EC-seeded scaffolds and control PG73 scaffolds alone [43]. Additionally, macrophage infiltration, typically observed in the later stages of the foreign body response, appeared to resolve more quickly for iPSC-EC-seeded scaffolds compared to scaffolds alone. Instead, the unexpected early increase in macrophages surrounding iPSC-EC-seeded scaffolds following implantation suggests altered tissue remodeling events. Macrophages have the capacity to influence multiple phases of the angiogenic process, including alterations of the local extracellular matrix and induction of endothelial cell migration and proliferation through angiogenic cytokine release [44].

Increased iPSC-EC engraftment in the first days post implantation coincides with heightened PlGF expression, a potent macrophage chemoattractant [45]. This enhanced macrophage presence at the implantation site, along with elevated expression of the classical angiogenic cytokine VEGF, may account for the increased density of large-diameter arterioles. Localization analysis of these cytokines revealed that these angiogenic factors originate from within the scaffold, possibly from engrafted iPSC-ECs and macrophages responding to the implant. Equally important to the therapeutic translation of these processes is the timing of cytokine release. Although enhanced VEGF expression in scaffolds alone is eventually achieved at similar expression levels to iPSC-EC-seeded scaffolds by day 9, levels at day 2 are significantly improved only in the presence of iPSC-ECs. The benefits of iPSC-EC-mediated increases in angiogenesis arise from this timely augmentation of blood perfusion immediately following implantation, in addition to the longevity of these effects even after iPSC-EC engraftment is no longer observed. Further research into identifying additional factors underlying this function is necessary for optimizing the angiogenic potential of future iPSC-EC scaffold therapies.

## Conclusion

The integration of biomaterials with iPSC-ECs is a highly promising and innovative approach for therapeutic angiogenesis. Optimization of these approaches for future therapy requires a comprehensive understanding of cell–material interactions and the resulting effects following implantation of these combined constructs. The findings of this study identify important scaffold design parameters which influence iPSC-EC behavior and function. These initial observations serve as preliminary criteria for developing candidate biomaterial platforms with enhanced capacity to support iPSC-EC engraftment and viability in vivo. Further understanding of the mechanisms underlying the combined effects of iPSC-ECs and their engraftment platforms will more readily lead to the development of enhanced iPSC-EC scaffold designs with clinical applications for therapeutic angiogenesis. The findings of this work help highlight this emerging cell–biomaterial field of regenerative medicine with significant implications for the treatment of ischemic injury.

## Additional files

Additional file 1: **Figure S1. **showing (**A**) representative SEM photographs of PG73 and PG55 scaffolds before and after soaking in PBS for 7 days, scale bar represents 10 μm. (**B**) Scaffold cross-section images for porosity analysis. *n* = 5 samples/group, scale bar represents 100 μm. (TIFF 19523 kb)

Additional file 2: **Figure S2. **showing (**A**) analysis of iPSC-EC infiltration into scaffolds, representative photographs of scaffold cross-sections stained with DAPI. *n* = 100 cells/scaffold, scale bar represents 100 μm. (**B**) Young’s modulus and (**C**) ultimate tensile strength (UTS) of PG73 scaffolds before and after wetting. *n* = 5 samples/group. (**D**) Degradation rate of PG73 scaffolds compared to PCL over 4 days in an accelerated Protease XIV degradation solution. *n* = 3 samples/group. (TIFF 12785 kb)

Additional file 3: **Figure S3.** showing (**A**) bioluminescence standard curve with upper limit of iPSC-EC concentration detection before IVIS camera saturation, inset. (**B**) Lower limit of iPSC-EC concentration detection after background/noise subtraction. *n* = 5 samples/group. (TIFF 44988 kb)

Additional file 4: **Figure S4.** showing (**A**) dedifferentiation of iPSC-ECs seeded on PG73 vs PCL scaffolds after 7 days culture *in vitro. Photographs* using (**B**) immunostains for CD31^+^ for endothelial phenotype and (**C**) vimentin^+^ for fibroblast phenotype. *p < 0.05. *n* = 3 samples/group, scale bar represents 40 μm. (TIFF 51787 kb)

## Abbreviations

CD31: Cluster of differentiation 31 (endothelial marker)
EGF: Epidermal growth factor
iPSC-EC: Induced pluripotent stem cell-derived endothelial cell
PCL: Polycaprolactone
PG73: PCL/gelatin (70:30 ratio)
PlGF: Placental growth factor
SEM: Scanning electron microscopy
SMC-α: Smooth muscle cell-actin
VEGF: Vascular endothelial growth factor
