# Supplementary figures and images for: Integration of induced pluripotent stem cell-derived endothelial cells with polycaprolactone/gelatin-based electrospun scaffolds for enhanced therapeutic angiogenesis
Source: Stem Cell Res Ther. 2018 Mar 21;9:70. doi: 10.1186/s13287-018-0824-2 (PMC5863387; doi:10.1186/s13287-018-0824-2)

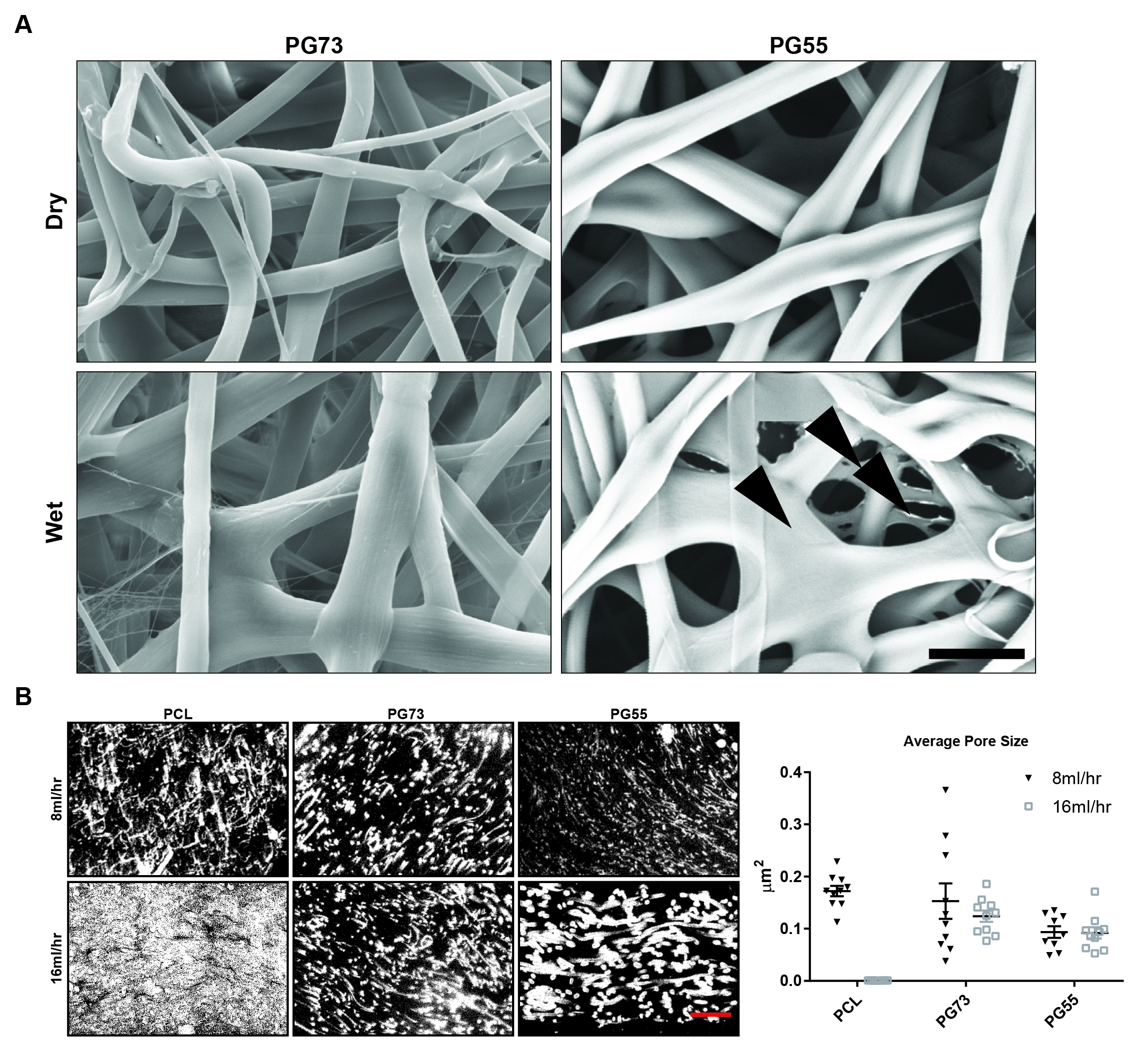

Supplement: Supplementary file 1 — Figure S1. showing (A) representative SEM photographs of PG73 and PG55 scaffolds before and after soaking in PBS for 7 days, scale bar represents 10 μm. (B) Scaffold cross-section images for porosity analysis. n = 5 samples/group, scale bar represents 100 μm. (TIFF 19523 kb) [file 13287_2018_824_MOESM1_ESM.tif]

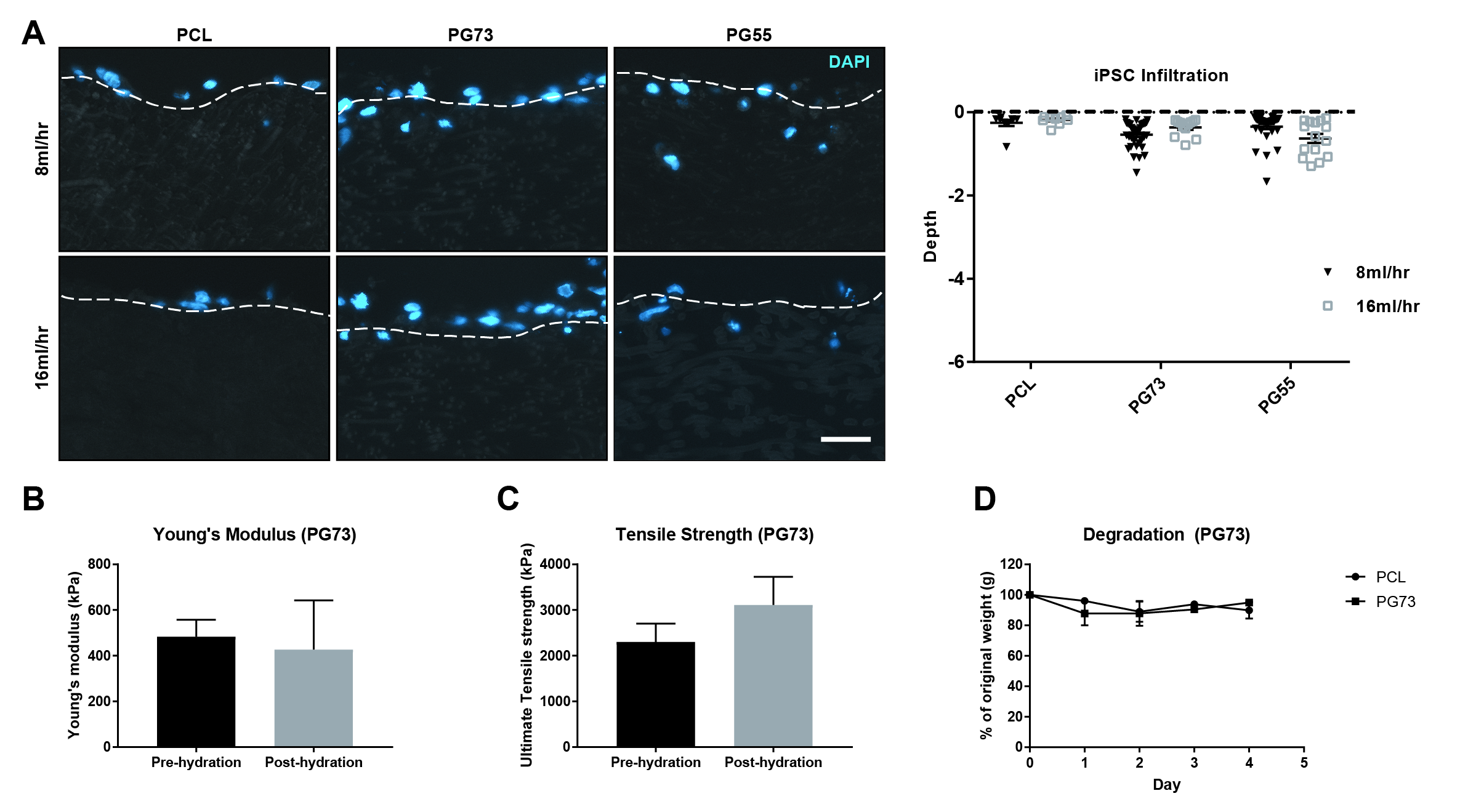

Supplement: Supplementary file 2 — Figure S2. showing (A) analysis of iPSC-EC infiltration into scaffolds, representative photographs of scaffold cross-sections stained with DAPI. n = 100 cells/scaffold, scale bar represents 100 μm. (B) Young’s modulus and (C) ultimate tensile strength (UTS) of PG73 scaffolds before and after wetting. n = 5 samples/group. (D) Degradation rate of PG73 scaffolds compared to PCL over 4 days in an accelerated Protease XIV degradation solution. n = 3 samples/group. (TIFF 12785 kb) [file 13287_2018_824_MOESM2_ESM.tif]

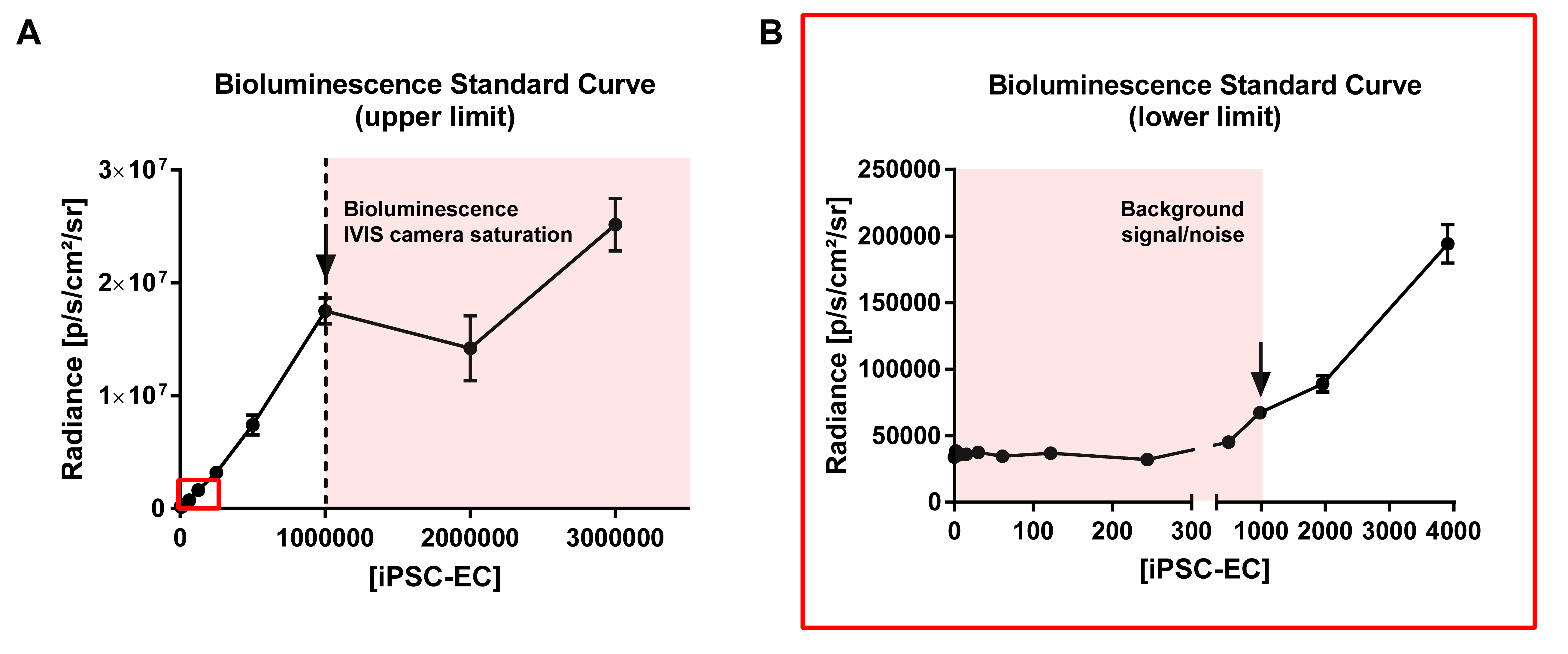

Supplement: Supplementary file 3 — Figure S3. showing (A) bioluminescence standard curve with upper limit of iPSC-EC concentration detection before IVIS camera saturation, inset. (B) Lower limit of iPSC-EC concentration detection after background/noise subtraction. n = 5 samples/group. (TIFF 44988 kb) [file 13287_2018_824_MOESM3_ESM.tif]

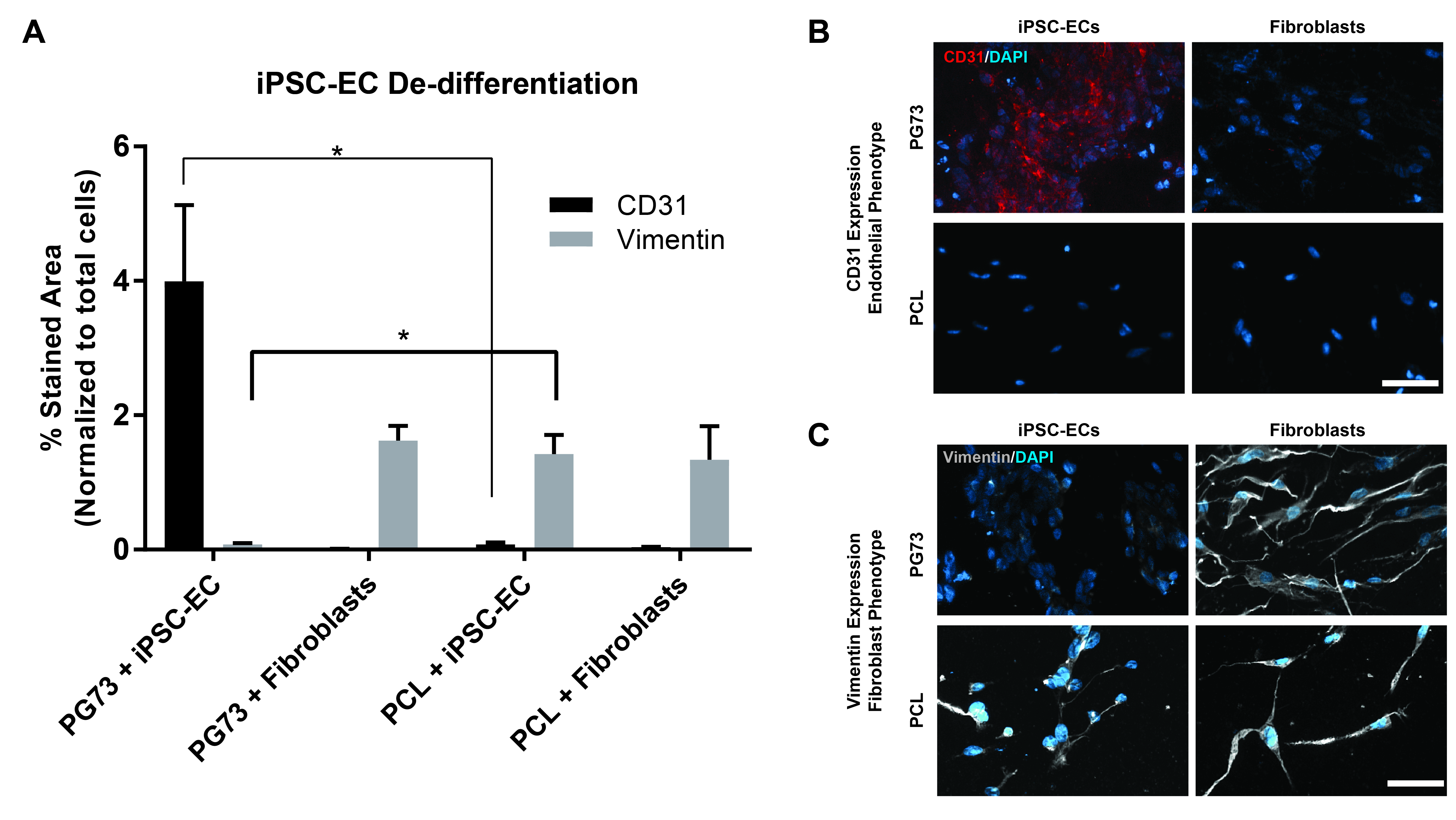

Supplement: Supplementary file 4 — Figure S4. showing (A) dedifferentiation of iPSC-ECs seeded on PG73 vs PCL scaffolds after 7 days culture in vitro. Photographs using (B) immunostains for CD31+ for endothelial phenotype and (C) vimentin+ for fibroblast phenotype. *p < 0.05. n = 3 samples/group, scale bar represents 40 μm. (TIFF 51787 kb) [file 13287_2018_824_MOESM4_ESM.tif]
